# Supplementary figures and images for: Social network community structure and the contact-mediated sharing of commensal E. coli among captive rhesus macaques (Macaca mulatta)
Source: PeerJ. 2018 Jan 17;6:e4271. doi: 10.7717/peerj.4271 (PMC5775753; doi:10.7717/peerj.4271)

A

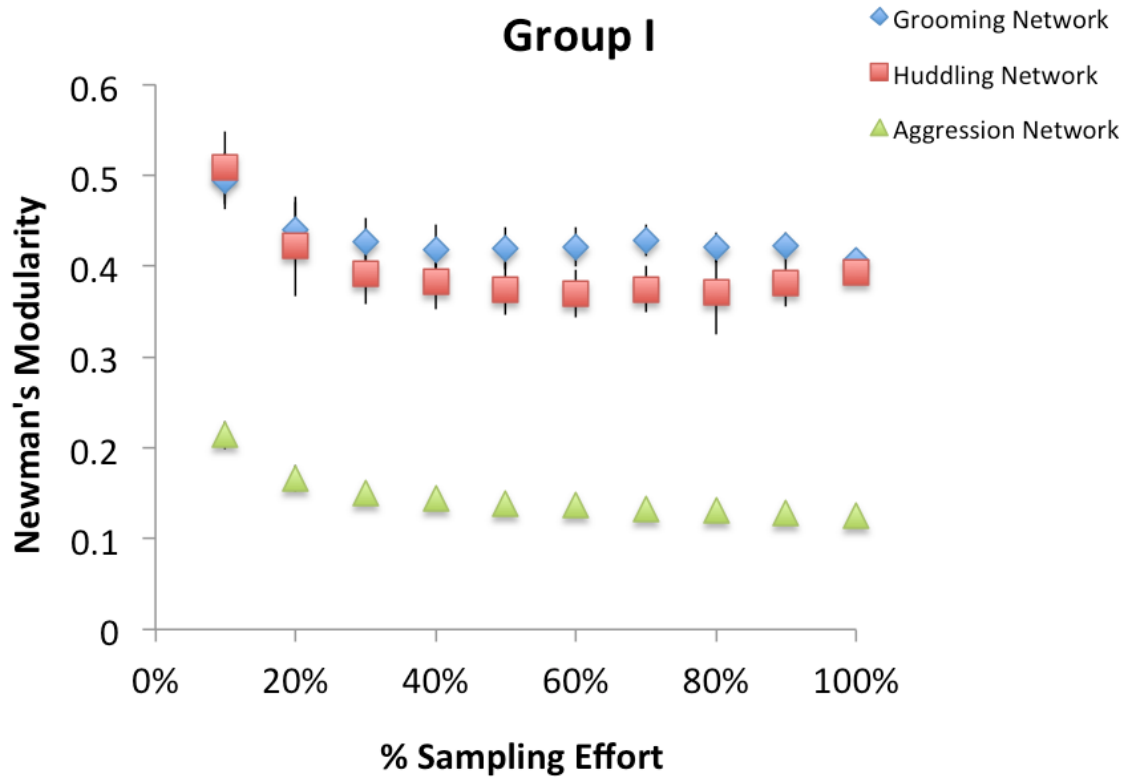

B

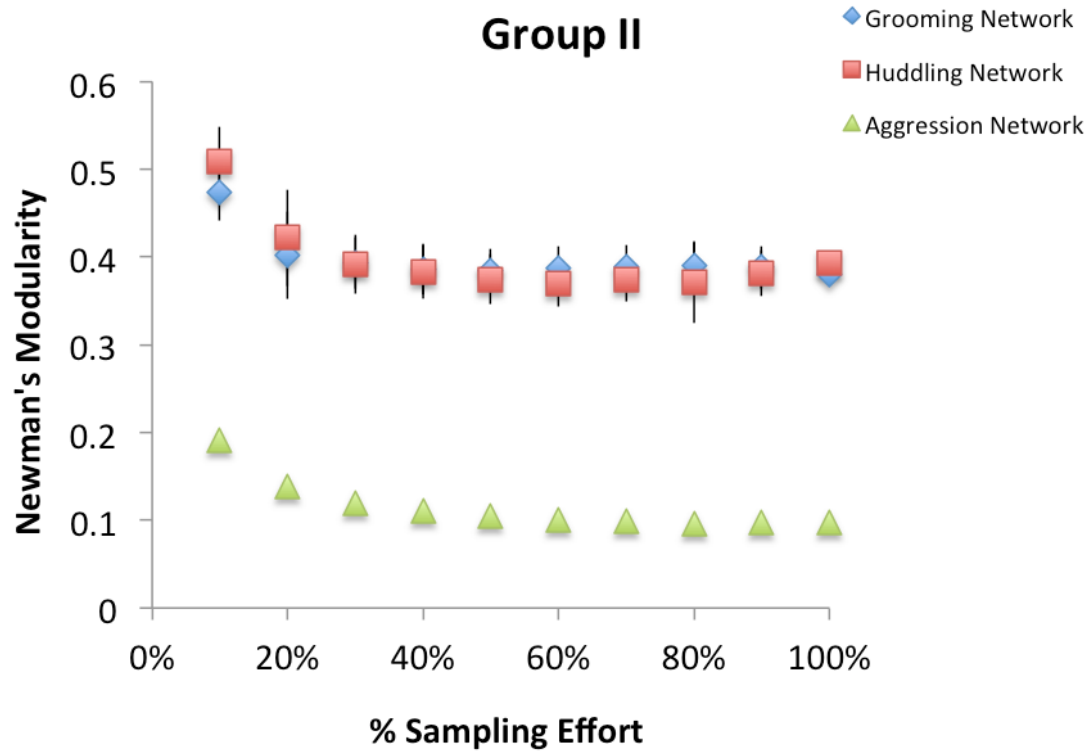

C

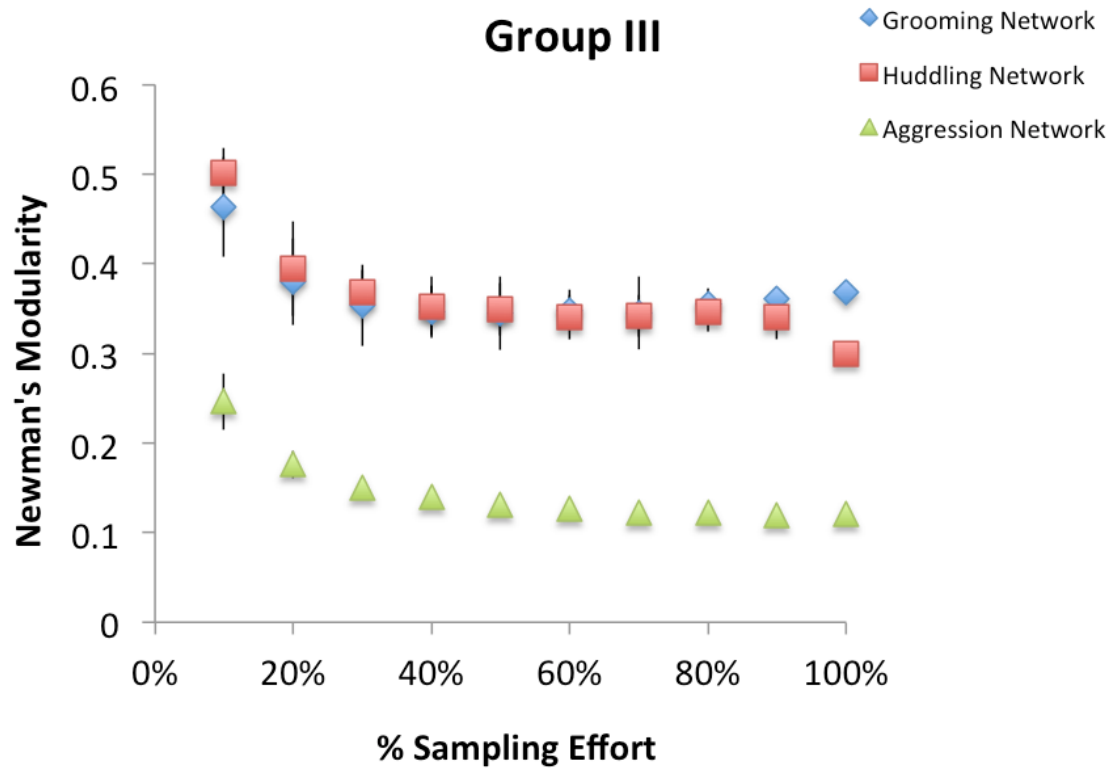

Supplement: Figure S1 — Data-points represent mean values computed from 1,000 networks (100 under each category of % sampling effort) permuted from each natural dataset, and error bars the standard deviations. Data-points at 100% sampling effort represent the modularity of the original network. [file peerj-06-4271-s001.pdf]
