# Supplementary material for: Social network community structure and the contact-mediated sharing of commensal E. coli among captive rhesus macaques (Macaca mulatta)
Source: PeerJ. 2018 Jan 17;6:e4271. doi: 10.7717/peerj.4271 (PMC5775753; doi:10.7717/peerj.4271)
Supplement: Table S1 [file peerj-06-4271-s002.docx]

| Network Measure | Description | Code Source |  |
| --- | --- | --- | --- |
|  | | | |
| Newman’s Modularity | The extent to which a network can be divided into communities of individuals which interact more among themselves (than expected by chance), than with members from another community | *Igraph* R package  (Csardi & Nepusz, 2006) |  |
| Mean degree | The average of the number of individuals to which each node (or individual) is connected to in the network | *Statnet* R package (Handcock et al., 2006) |  |
| Density | The number of observed edges in the network divided by the total number of possible edges (n^2^ – n, with ‘n’ being the number of nodes) | *Statnet* R package (Handcock et al., 2006) |  |
