# Supplementary material for: Social network community structure and the contact-mediated sharing of commensal E. coli among captive rhesus macaques (Macaca mulatta)
Source: PeerJ. 2018 Jan 17;6:e4271. doi: 10.7717/peerj.4271 (PMC5775753; doi:10.7717/peerj.4271)
Supplement: Table S4 [file peerj-06-4271-s005.docx]

| **Multivariate Model** |  | **Group I** | | **Group II** | | **Group III^a^** | |
| --- | --- | --- | --- | --- | --- | --- | --- |
| E. coli % similarity ~ |  | B | p  (α = 0.01) | B | p  (α = 0.01) | B | p  (α = 0.01) |
| Grooming freq. + |  | -7.98 | 0.10 | 0.40 | 0.94 | -5.48 | 0.36 |
| Huddling freq. + |  | 2.18 | 0.77 | -13.14 | 0.13 | 2.20 | 0.70 |
| Aggression freq. |  | 4.30 | 0.33 | 7.47 | 0.02 | 0.09 | 0.98 |
